# New application of strontium isotopes reveals evidence of limited migratory behaviour in Late Cretaceous hadrosaurs

**DOI:** 10.1098/rsbl.2019.0930

**Published:** 2020-03-04

**Authors:** David F. Terrill, Charles M. Henderson, Jason S. Anderson

**Affiliations:** 1 Department of Geoscience, University of Calgary, Calgary, Alberta, Canada; 2 Department of Comparative Biology and Experimental Medicine in the Faculty of Veterinary Medicine, University of Calgary, Calgary, Alberta, Canada

**Keywords:** hadrosaur, migration, strontium isotopes, geochemistry, Late Cretaceous

## Abstract

Dinosaur migration patterns are very difficult to determine, often relying solely on the geographical distribution of fossils. Unfortunately, it is generally not possible to determine if a fossil taxon's geographical distribution is the result of migration or simply a wide distribution. Whereas some attempts have been made to use isotopic systems to determine migratory patterns in dinosaurs, these methods have yet to achieve wider usage in the study of dinosaur ecology. Here, we have used strontium isotope ratios from fossil enamel to reconstruct the movements of an individual hadrosaur from Dinosaur Provincial Park in Alberta, Canada. Results from this study are consistent with a range or migratory pattern between Dinosaur Provincial Park and a contemporaneous locality in the South Saskatchewan River area, Alberta, Canada. This represents a minimum distance of approximately 80 km, which is consistent with migrations seen in modern elephants. These results suggest the continent-wide distribution of some hadrosaur species in the Late Cretaceous of North America is not the result of extremely long-range migratory behaviours.

## 1. Introduction

Since it was first suggested by von Huene [1], the idea of dinosaur migration has been the subject of much debate. Traditional ideas of migration were centred around the distribution of fossil taxa which, in many cases, span areas of thousands of kilometres [1,2]. This approach is flawed however, as some species may have had a wide species distribution unrelated to migration, such as the modern cougar (*Puma concolor*), which is found from the Yukon of Canada to the southern tip of Argentina and Chile [3]. The debate about possible dinosaur migration achieved new prominence with the discovery of dinosaur trackways at polar latitudes [4], which have since been corroborated by skeletal dinosaur finds in Antarctica [5,6], Alaska [7], New Zealand [8] and Russia [9]. These high latitude faunas raised questions surrounding the ability of dinosaurs to survive polar winters, resulting in competing hypotheses of migration versus overwintering [2,7]. This has led to debate about the biomechanical ability of dinosaurs to migrate, metabolism, endothermy and bone structure [2,7,9–12]. Most recent work suggests that dinosaurs did possess the necessary adaptations to overwinter in the much milder Cretaceous climate [2,7,8,9,11,12]; however, this does not preclude the possibility that some dinosaurs may have migrated within both polar and more temperate regions [2,7].

One approach to this problem is to use the concept of isoscapes, which can be described as spatially driven changes in the availability of different isotopes over broad geographical areas (or landscapes) [13]. In recent years, some workers have attempted to measure migration using stable isotopes of carbon and oxygen preserved in the enamel of dinosaur teeth [10,14]. By contrast, our study uses the ratio between the radiogenic strontium 87 isotope and the stable strontium 86 isotope (^87^Sr/^86^Sr). Unlike carbon and oxygen isotope ratios, which vary depending on a variety of environmental conditions [10,14], strontium isotope ratios in the enamel of terrestrial vertebrates are generally reflective of diet and freshwater consumption, as any mass fractionation that has occurred is normalized according to an exponential mass fractionation law during analysis [15]. Strontium isotope ratios in animals and plants reflect values in nearby freshwater sources [15,16] and soils [17], which are primarily derived from bedrock weathering [17]. As a result, strontium isotope signatures can vary between nearby river basins, as documented in modern Canadian river basins [18], and can therefore be used to infer the presence of an individual organism in a specific geographical region. While strontium has been used to study food web patterns in fossil materials for more than half a century [19], it was not until 1985 when strontium isotopes began to be used in human migration studies [20]. Since then, strontium isotope ratios have been increasingly used in migration studies in archaeology and palaeontology [17,21–24] including habitat preference studies of Mesozoic aquatic reptiles [25,26], but applications of this methodology to terrestrial animals outside the Quaternary are largely absent.

For this study, we applied a strontium isotope approach to investigate migratory behaviours in hadrosaurs from the Late Cretaceous of Alberta, Canada. Hadrosaurs were widespread during the Late Cretaceous of North America, with some genera ranging from Mexico to Alaska [2], and the genus *Edmontosaurus* confirmed to range over 2600 km [2]. Hadrosaurs are therefore considered as one of the more likely dinosaur groups to undertake long-distance migrations. They are also known to have rapidly grown new teeth (every 6–12 months, with replacement rates of approx. 2 months [27]), allowing an individual to possess teeth formed in different localities, with each tooth reflecting the ^87^Sr/^86^Sr value of the locality in which it formed. These factors make hadrosaurs an ideal target for dinosaur migration studies.

**Figure 1.**
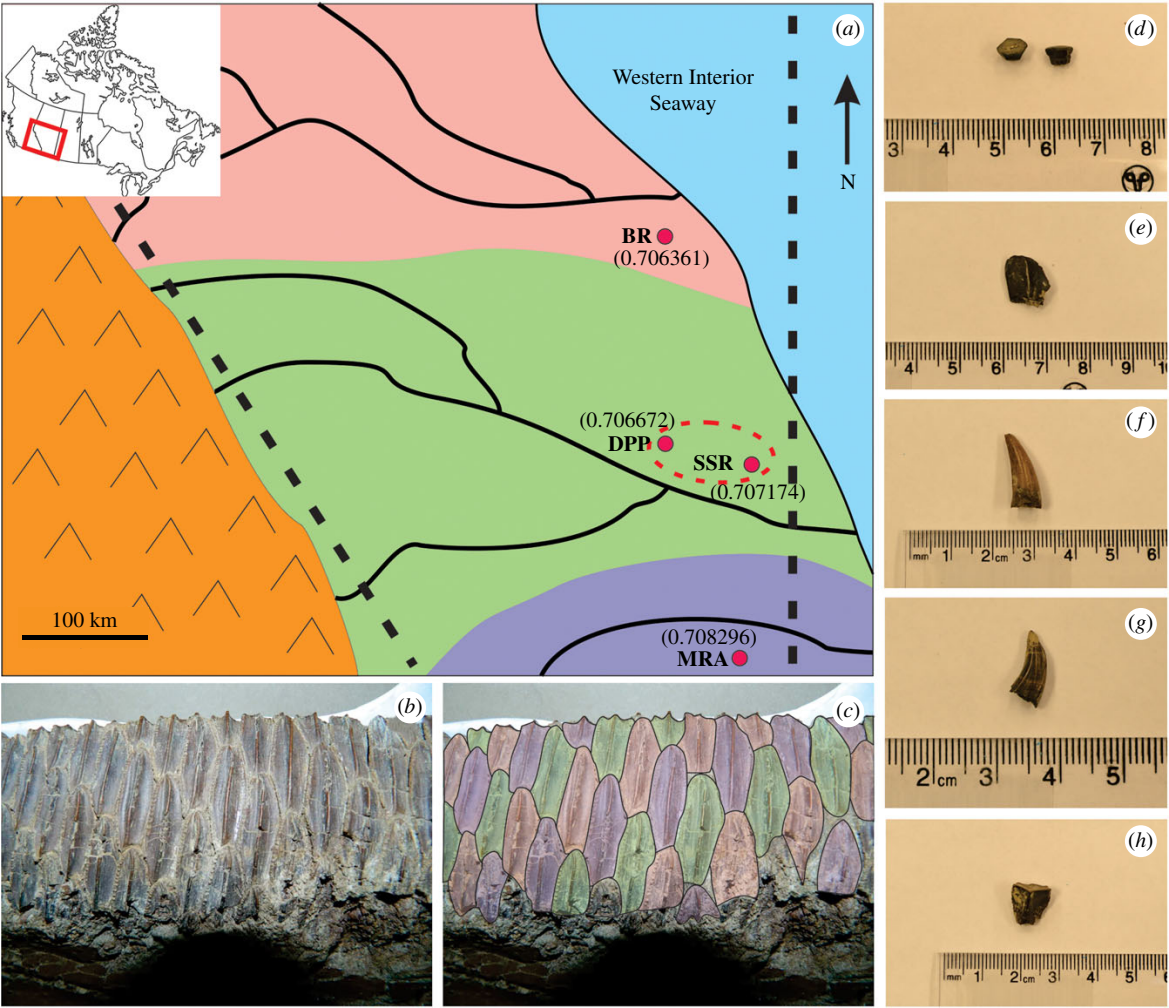
Palaeogeographic reconstruction of Western Canada 75 Ma, with the current modern map area indicated on the inset (*a*). This diagram depicts three separate hypothetical ancient river basins, each with a corresponding strontium isotope signature as signified by the colours purple, green and pink. These isotopic signatures are hypothesized to be recorded in newly formed teeth in a hadrosaur, as depicted in (*b,c*). Strontium isotope ratios (means shown in brackets) from each studied locality (DPP, Dinosaur Provincial Park; SSR, South Saskatchewan River; BR, Battle River; MRA, Milk River area) were determined by analysing enamel from vertebrates confined to a single river, including freshwater rayfish teeth (*d*), gar-pike scales (*e*) and crocodile teeth (*f*), which were then compared with animals with greater potential range such as carnivorous and herbivorous dinosaurs (*g,h*, respectively). The dashed red circle depicts the simplest solution for the range of the individual hadrosaur in this study as supported by the data.

To determine if strontium isotope data in hadrosaur teeth indicate migratory behaviour, it was necessary to compare the results against those from presumed non-migratory taxa. These taxa included freshwater fish, crocodiles and small theropods. These non-migratory taxa were used to estimate the range of strontium isotope ratios that were present in a given locality during the Late Cretaceous (figure 1).

## 2. Material and methods

Samples of microvertebrate material from a variety of approximately time-equivalent localities were obtained from the Royal Tyrrell Museum, including samples from Dinosaur Provincial Park, the South Saskatchewan River, Milk River and Battle River areas. Most material was from the Dinosaur Park Formation (DPF), with the exception of some samples from the Dinosaur Provincial Park and Milk River areas. Additional samples from the Dinosaur Provincial Park area were obtained through field collections. It was important to minimize the effects of possible changes in the environmental strontium isotope content that can occur over geological time, so fossil localities were chosen for their approximate time equivalency (see electronic supplementary material, for geological details).

Specimens analysed in this study represent a variety of taxa, including hadrosaurs, small theropods, crocodilians, *Myledaphus* (freshwater guitarfish) and gar. Most isotope samples were collected from the enamel or enameloid of fossil teeth or scales, as dentine is more susceptible to diagenetic alteration [21]; however, some dentine samples were also collected to evaluate the possibility of diagenetic overprinting in the enamel samples (see table 1 for a complete summary). Enamel samples from 17 teeth from an individual hadrosaur specimen from the DPF in Dinosaur Provincial Park were also examined, along with a few dentine samples. Finally, two teeth and a bone fragment were analysed for strontium concentrations along with a modern deer tooth, using an isotope dilution. This was done as a further means of assessing possible diagenetic contamination. Details on how samples were prepared and analysed can be found in the electronic supplementary material.

**Table 1.** Summary of all collected data from enamel/enameloid sources. The analytical uncertainty is 20 ppm at *k* = 2. Locations are indicated by the same abbreviations as in figure 1. Formations are indicated by the same abbreviations as in the text. Samples numbers J-1 to J-17 are from an individual hadrosaur, while all other samples are from isolated elements.

sample	^87^Sr/^86^Sr	taxon	location	formation
T1	0.706673	theropod	DPP	DPF
T2	0.706593	theropod	DPP	DPF
T3	0.706679	theropod	DPP	DPF
C1	0.706711	crocodile	DPP	DPF
C2	0.706643	crocodile	DPP	DPF
C3	0.706692	crocodile	DPP	DPF
C4	0.706648	crocodile	DPP	DPF
M1	0.706620	*Myledaphus*	DPP	DPF
M2	0.706651	*Myledaphus*	DPP	DPF
M3	0.706651	*Myledaphus*	DPP	DPF
M4	0.706761	*Myledaphus*	DPP	DPF
G1	0.706865	gar	DPP	DPF
G2	0.706716	gar	DPP	DPF
G4	0.706707	gar	DPP	DPF
146-1	0.706643	crocodile	DPP	DPF
146-2	0.706676	crocodile	DPP	DPF
146-3	0.706706	crocodile	DPP	DPF
146-4	0.706592	crocodile	DPP	DPF
146-6	0.706515	gar	DPP	DPF
146-7	0.706696	gar	DPP	DPF
H1	0.706818	hadrosaur	DPP	DPF
H2	0.706672	hadrosaur	DPP	DPF
H3	0.706278	hadrosaur	DPP	DPF
H4	0.706959	hadrosaur	DPP	DPF
J-1	0.706739	hadrosaur	DPP	DPF
J-2	0.706901	hadrosaur	DPP	DPF
J-3	0.706861	hadrosaur	DPP	DPF
J-4	0.706923	hadrosaur	DPP	DPF
J-5	0.706813	hadrosaur	DPP	DPF
J-6	0.7068	hadrosaur	DPP	DPF
J-7	0.706885	hadrosaur	DPP	DPF
J-8	0.706975	hadrosaur	DPP	DPF
J-9	0.706759	hadrosaur	DPP	DPF
J-10	0.706932	hadrosaur	DPP	DPF
J-11	0.707061	hadrosaur	DPP	DPF
J-12	0.707059	hadrosaur	DPP	DPF
J-13	0.70694	hadrosaur	DPP	DPF
J-14	0.706917	hadrosaur	DPP	DPF
J15	0.706936	hadrosaur	DPP	DPF
J-16	0.706855	hadrosaur	DPP	DPF
J-17	0.707044	hadrosaur	DPP	DPF
100-1	0.707293	*Myledaphus*	DPP	OMF
100-2	0.707227	*Myledaphus*	DPP	OMF
100-3	0.707366	*Myledaphus*	DPP	OMF
100-4	0.707246	*Myledaphus*	DPP	OMF
100-5	0.707752	crocodile	DPP	OMF
100-6	0.707558	gar	DPP	OMF
100-7	0.707293	theropod	DPP	OMF
Br-1.1	0.706373	*Myledaphus*	BR	DPF
Br-1.2	0.706109	*Myledaphus*	BR	DPF
Br-1.3	0.706383	*Myledaphus*	BR	DPF
Br-1.4	0.706605	*Myledaphus*	BR	DPF
Br-2	0.706356	crocodile	BR	DPF
Br-3	0.706409	crocodile	BR	DPF
Br-4.1	0.706148	*Myledaphus*	BR	DPF
Br-4.2	0.706176	*Myledaphus*	BR	DPF
Br-4.3	0.706224	*Myledaphus*	BR	DPF
Br-5.1	0.706438	crocodile	BR	DPF
Br-5.2	0.706751	crocodile	BR	DPF
HAS-1	0.708269	theropod	MRA	OMF
HAS-2	0.708347	theropod	MRA	OMF
HAS-3	0.708323	gar	MRA	OMF
HAS-4	0.708289	gar	MRA	OMF
HAS-5	0.708254	gar	MRA	OMF
SSR-1	0.707399	*Myledaphus*	SSR	DPF
SSR-2	0.707306	*Myledaphus*	SSR	DPF
SSR-3	0.707183	*Myledaphus*	SSR	DPF
SSR-4	0.707279	crocodile	SSR	DPF
SSR-5	0.707321	crocodile	SSR	DPF
SSR-6	0.707053	theropod	SSR	DPF
SSR-7	0.706923	crocodile	SSR	DPF
SSR-8	0.706931	crocodile	SSR	DPF
SSR-10	0.706991	crocodile	SSR	DPF
SSR-12	0.707041	*Myledaphus*	SSR	DPF
SSR-13	0.707108	crocodile	SSR	DPF
SSR-14	0.707506	*Myledaphus*	SSR	DPF
SSR-15	0.707275	*Myledaphus*	SSR	DPF

## 3. Results and discussion

Chemical information, including strontium isotope ratios, is at risk of being altered after burial, primarily through contact with post-burial fluids. For this study, isotope data were primarily acquired from enamel, owing to that tissue's much higher resistance to post-burial alterations. Steps were also taken to reduce or eliminate any residual diagenetic strontium. We also performed a Sr concentration analysis on two hadrosaur teeth to check for diagenetic signals (see electronic supplementary material, figure S1). This analysis showed possible enrichment in total strontium in all tissues, with much higher values in dentine.

Five dentine samples were collected from two hadrosaur teeth and one fish scale at DPP and were found to preserve near identical Sr isotope ratios (electronic supplementary material, figure S2). This lack of variability suggests a single diagenetic event, which overprinted the original ratios in these fossils. By contrast, enamel samples maintain a significant variance, with enamel and dentine samples from the same teeth preserving divergent isotope ratios. While enamel Sr concentrations may be elevated, this greater variability in isotope ratios suggests that enamel does preserve at least some of the original biogenic strontium signature.

**Figure 2.**
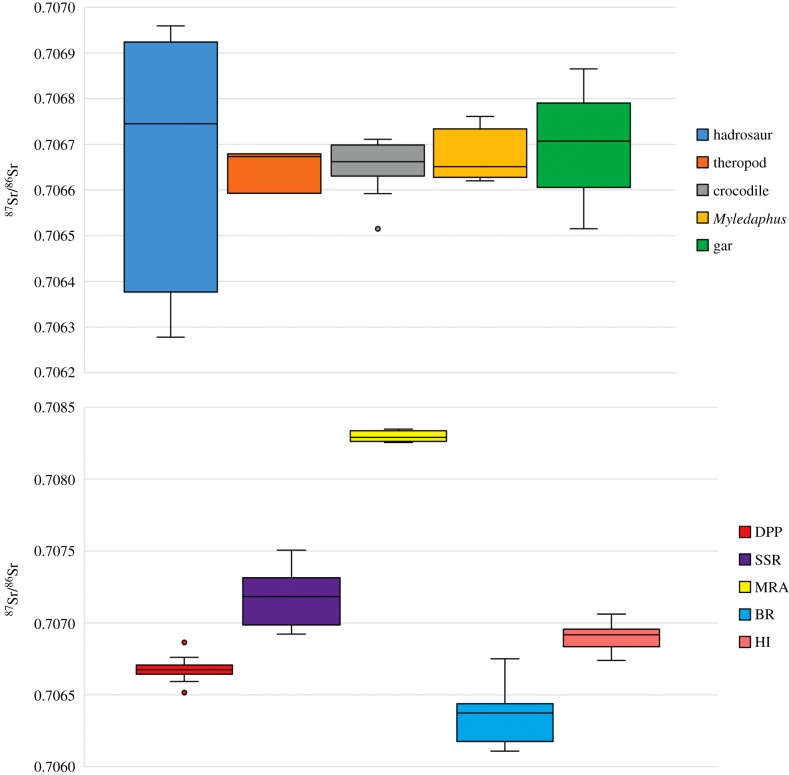
Summary of isotope data collected in this study. The upper chart shows the data ranges for different taxa collected from the Dinosaur Park Formation at Dinosaur Provincial Park, while the lower chart contains data from presumed non-migratory taxa from a variety of approximately co-eval localities alongside data collected from the hadrosaur individual (HI) from the DPF at Dinosaur Provincial Park.

The results are summarized in table 1 and figure 2. Material from the DPF at Dinosaur Provincial Park show a relatively narrow range of ^87^Sr/^86^Sr ratios with the only exception being the hadrosaur specimens, which show a much wider range of values (figure 2), a result consistent with hadrosaurs being more mobile. Other taxa in this study are interpreted as non-migratory, owing to the narrower range of strontium isotope values and their general biology (smaller size, mobility tied to waterways, etc.). Presumed non-migratory taxa from other localities produced distinctive ranges of Sr isotope ratios; the SSR locality preserved slightly more radiogenic Sr ratios while the MRA locality preserved much more radiogenic ratios, and the BR locality preserved a significantly lower radiogenic range. To these results, we compared the Sr isotope ratio range preserved in the enamel of the individual hadrosaur from DPP. The jaw presents an overall range of values that overlaps with non-migratory values found in both the DPP and SSR localities, with a mean value close to the midpoint between the mean values at DPP and SSR. A Tukey statistical test confirmed the individual preserved a unique signature from these localities (electronic supplementary material, figure S3). In total, these data support a limited hadrosaur migration distance or large home range between the Dinosaur Provincial Park and South Saskatchewan River areas while discounting any migratory link to the Milk River area.

The total distance between the Dinosaur Provincial Park and South Saskatchewan River localities is approximately 80 km, a distance that is relatively comparable to migrations covered by modern elephants in Africa [28] or the home ranges of elephants in Borneo [29], which are among the best modern analogues for hadrosaurs on the basis of similarities in mass and presumed diet. There is little palaeo-environmental difference between these two localities; both are located in the Dinosaur Park Formation, which represents a coastal floodplain environment dominated by meandering river systems [30]. The only difference between the localities is the relative proximity to the coastline (with the South Saskatchewan River locality nearer the coast). Using the current results, it is not yet possible to determine if this is a seasonal migration pattern, or simply represents the natural range of the individual. Given the environmental similarities, it is unlikely that seasonal weather or food shortages would be the impetus for such a migration; however, we cannot rule out migratory behaviour for the purposes of breeding.

Given the abundance of hadrosaur fossils collected in the DPF [31,32], biological productivity rates must have been quite high all year to support a large population of resident hadrosaurs. While most other herbivores found in the DPF consumed very low browse, hadrosaurs were capable of consuming higher elevation plants, being able to reach vegetation as high as 5 m [33,34]. This ability to exploit different vegetation sources may have been key to the success of hadrosaurs in the area, allowing them to avoid large-scale migrations.

It has previously been suggested that arctic hadrosaurs migrated hundreds or thousands of kilometres every year in a fashion similar to modern caribou [7,35,36]. The results of this study do not support this model, and corroborate a non-migratory model based on biomechanical grounds [12]. Our data show no overlap between the hadrosaur individual from Dinosaur Provincial Park and the southern Milk River locality or more northern Battle River locality, suggesting no significant southerly or northerly migration. While we cannot rule out larger migrations to the west because of the absence of other locality data, the simplest solution supported by the data is that of a smaller migratory path or large home range in southern Alberta, well short of the thousands of kilometres that would have been necessary for dinosaurs on the North Slope of Alaska to reach the milder winter conditions that would have been found in Alberta during the Late Cretaceous. This is further supported by strontium isotope data from a previous study on stratigraphically younger hadrosaurs from the Prince Creek Formation of Alaska, in which values between 0.7081 and 0.7092 were obtained [37]. While we should be cautious in ascribing limited migratory behaviour to all species of hadrosaurs found in all regions of North America, our conclusions are in agreement with recent work suggesting dinosaurs were limited in their migrations and likely overwintered in the ancient arctic [2,8,10,11,12]. Our conclusions are also in agreement with those of Fricke *et al*. [10], who used oxygen and carbon isotopes to evaluate migratory behaviours (or lack thereof) in hadrosaurs and concluded that large continent-scale migrations were unlikely.

The use of strontium isotopes in this study shows considerable utility in determining migratory and ranging behaviours in extinct dinosaurs. While data from a larger number of localities could better constrain this movement, we are confident that our method suggests a minimum 80 km range for hadrosaurs living in southern Alberta during the Late Cretaceous. This method could be extended to other possibly migratory taxa, particularly ceratopsians, which are interpreted to have travelled in very large herds during this time [38,39]. Using strontium isotopes, we may be able to quantify migratory behaviours, thereby gaining a better understanding of the ecology of extinct terrestrial organisms and how they moved through their respective environments.

## Supplementary Material

Supplemental Geological Information and Methods

## Data accessibility

All data used in this study can be accessed via the Dryad Digital Repository: https://dx.doi.org/10.5061/dryad.x95x69pf0 [40].
