# Supplementary material for: New application of strontium isotopes reveals evidence of limited migratory behaviour in Late Cretaceous hadrosaurs
Source: Biol Lett. 2020 Mar 4;16(3):20190930. doi: 10.1098/rsbl.2019.0930 (PMC7115185; doi:10.1098/rsbl.2019.0930)
Supplement: Supplemental Geological Information and Methods [file rsbl20190930supp1.docx]

**Supplemental Information**

**Methods**

To collect material from each specimen, the outer surface of each fossil in this study was removed using a carbide or diamond tipped Dremel to minimize the impact of contamination and diagenesis. This is estimated to represent just a few microns, although exact depths are not known. Material was then collected using individually cleaned Dremel bits, with sample weights of at least 1.2 mg (with most samples being between 2 and 3 mg). Each collected sample was then pretreated using acetic acid to remove any potential diagenetic carbonate. Samples were reacted three times in 0.5 ml of 0.1N acetic acid for 20 minutes at a time, followed by a 3-minute centrifuge and removal of the acid via pipette. After the third acetic acid treatment, samples were rinsed using Milli-Q water. Finally, samples were digested in a solution of 0.5 ml 5.0M nitric acid and 30 microlitres of peroxide. After digestion, samples were dried under a heatlamp in preparation for the ion exchange.

For the ion exchange, columns were filled with approximately 100 microlitres of Eichrom Sr-Spec resin. The resin was rinsed with 5 ml of MQ water, then preconditioned with 0.5 ml of 3.0M nitric acid. Samples were then re-dissolved in 0.5ml of 3.0M nitric acid before being loaded into the columns. The samples were then rinsed twice in 1ml of 3.0M nitric acid, before undergoing elution using 0.5 ml of MQ water.

To determine Sr concentrations, we collected enamel, dentin and bone samples from two hadrosaur teeth from the individual in this study, along with an enamel and dentin sample from a modern deer tooth. Sampling techniques were identical to those used to for isotope analysis. Preparation techniques were also identical with one major difference; samples were ‘spiked’ with an additional strontium solution prior to digestion. This solution was of known concentration and isotopic composition, with an enriched ^84^Sr content. ^84^Sr is a rare isotope in nature, allowing for easy detection through mass spectrometry. By measuring the ^84^Sr/^86^Sr content of a spiked sample, we were able to calculate the relative contributions of strontium provided by the spiked solution and the enamel/dentin/bone sample. As the mass of strontium added via the spike is known, we could then calculate the mass of strontium in each tooth sample, which in turn allowed for a calculation of the Sr concentration. This technique is commonly known as an isotope dilution procedure.

All measurements were made using the Triton model Thermal Ionization Mass Spectrometer (TIMS) at the Department of Physics and Astronomy, University of Calgary, which was calibrated using the NIST 987 SRM standard. The long term results and uncertainties for NIST 987 SRM measurements with this equipment is 0.710243, with a 2 SD value of 0.000012 and 2 SE value of 0.000002. No corrections were applied to the data due to deviations from the accepted NIST 987 SRM value of 0.710248 [41]

**Geology**

All fossils examined in this study were sourced either from the Dinosaur Park Formation or the underlying Oldman Formation in South-eastern Alberta, Canada. Both formations belong to the Belly River Group, also known as the Judith River Group, and are Late Campanian in age. Formation ages have been well established using a mix of radiogenic techniques, such as Ar-Ar analysis, and palynology biostratigraphy [30,42]. Both formations represent terrestrial ecosystems and are dominated by fluvial deposits and flood deposits. In all localities in this study, the palaeolatitude is believed to be several degrees further north, with the Dinosaur Provincial Park locality estimated to be between 57 and 58 degrees N, and all other localities within 1 degree of this latitude. This places these localities just south of the arctic circle during the Late Cretaceous [43].

One of the major differences between the Dinosaur Park Formation and Oldman Formation is the petrology. All petrological data presented here are from Eberth and Hamblin [30]. The Oldman Formation contains significant amounts of quartz grains (20-60%) and reworked carbonate grains (up to 15%), lacks extra-formational clasts, and contains low amounts of volcanic lithics (~2%). In contrast, the Dinosaur Park Formation contains relatively low amounts of quartz grains (20-30%) and reworked carbonate grains, contains extra-formational clasts, and significant amounts of volcanic lithic fragments (up to 10%). This is proposed to be the result of different sediment sources. The Oldman depositional lobe deposited sediments in a north-easterly direction, originating primarily in southern British Columbia and northern Montana [44]. In contrast, the Dinosaur Park depositional lobe originated in the north-west part of Alberta, transporting sediments toward the south-east and gradually expanding toward the south-eastern portion of the province at a rate of approximately 130-140 km per million years [44]. This change in depositional pattern is suggested to be tectonic in origin, resulting from differential loading and crustal rebound following orogenic thrusting in the Canadian Cordillera [30,44].

The Oldman and Dinosaur Park formations are separated by a diachronous facies boundary. The boundary occurs earlier in more northern localities, leading to fossil material collected from the upper Oldman Formation in the Milk River area being approximately coeval with material collected from the lower Dinosaur Park Formation at Dinosaur Provincial Park [30] (See figure below). The diachronous nature of the contact has traditionally been identified by depositional models and radiometric dates, but has also been recently supported by palynological analysis [42]. It is for this reason that we have compared the results obtained from fossil material in the upper Oldman Formation in the Milk River area to the hadrosaur individual analyzed in this study.

The farthest north locality included in this study is the Battle River site. As mentioned earlier, this material comes from the collections of the Royal Tyrrell Museum. While the locality was identified as Dinosaur Park Formation by museum staff, formation boundaries were not present at the site, nor have any dateable ash beds been found. As such, the precision of the locality’s stratigraphic position cannot be determined beyond the formation in which it was found.

Figure S1: Simplified stratigraphy of the Judith River Group in southern Alberta, modelled from Eberth and Hamblin [30]. Orange represents the Oldman Formation, yellow the Dinosaur Park Formation, and brown the Bearpaw Formation. Grey layers represent volcanic ashes, which have been radiometrically dated using Ar-Ar. Stars are used on the diagram to denote approximate stratigraphic positioning of collection sites in these areas. The green star represents the individual Hadrosaur, while the pink stars represent microvertebrate fossil localities.

**Diagenesis**

The total concentration of strontium in a fossil can be used to detect the presence of diagenetic strontium. While concentrations of natural strontium may vary depending on several factors, including habitat and diet, many studies show common Sr concentrations of less than 50 ppm up to around 200 ppm in modern organisms [16,45,46]. Here we see a similar result in our modern deer tooth, as both enamel and dentin samples returned a value of just under 200 ppm. In contrast, enamel from the hadrosaur in this study returned concentrations of between 709 and 960 ppm, while dentin and bone samples were further enriched to between 1159 and 1302 ppm (see Table S1). These levels of enrichment suggest that some of the strontium signal is likely to be diagenetic, however enamel has taken up far less additional strontium than bone and dentin. It is also difficult to ascertain the degree to which strontium preserved in the fossil is diagenetic, as we are dealing with extinct animals whose habitat may have provided above average dietary strontium. Their physiology may have also allowed for higher levels of strontium uptake as well, as we are left only with distant relatives to which we can compare to. Some studies suggest modern archosaurs such as modern warblers, rheas and crocodiles may have relatively low Sr concentrations (<100 ppm)[16,47], while others suggest much higher values are possible in both modern crocodiles (121-923 ppm) [48] and modern egg laying chicken bones (>800 ppm) [49], values which are comparable to the hadrosaur enamel values in this study. Another study analyzing strontium isotopes in a range of fossil archosaurs also concluded that high concentrations of strontium in sauropod teeth (764+/-294 ppm) were not significantly elevated due to diagenesis [27].

While we must be aware of possible diagenetic contamination, we still interpret the data in this study as preserving at least some of the original biological signature, due to the greater variability of isotope ratios seen in hadrosaurs when compared to other taxa as well as much greater variability seen in enamel isotope ratios in comparison to dentin. Dentin isotope ratios are also very close to the mean ratio detected in enamel samples in non-migratory taxa in the Dinosaur Park Formation at DPP, suggesting that diagenesis likely occurred soon after burial, and reflected local environmental ratios. This is further supported by isotope ratios from the Oldman Formation at DPP, in which enamel Sr isotope ratios preserved a much more radiogenic signature despite occurring just a few meters (10-20m) below the DPF materials used in this study. A dentin sample from the OMF locality also preserved a more radiogenic signature than those from the DPF, supporting the hypothesis that diagenesis in these sediments occurred relatively quickly (~100 ky), and at relatively shallow depths.

| Sample Number | Tissue Type | Sr concentration (ppm) |
| --- | --- | --- |
| J-18.1 | Enamel | 959 |
| J-18.2 | Enamel | 800 |
| J-18.3 | Dentin | 1221 |
| J-18.4 | Dentin | 1159 |
| J-19.1 | Enamel | 709 |
| J-19.2 | Enamel | 909 |
| J-19.3 | Dentin | 1185 |
| J-19.4 | Dentin | 1302 |
| J-20 | Bone | 1297 |
| DT-1 | Enamel | 194 |
| DT-2 | Dentin | 183 |

Table S1: Strontium concentration analyses of two hadrosaur teeth (J-18 and J-19), hadrosaur bone (J-20), and two modern deer teeth (DT-1 and DT-2).


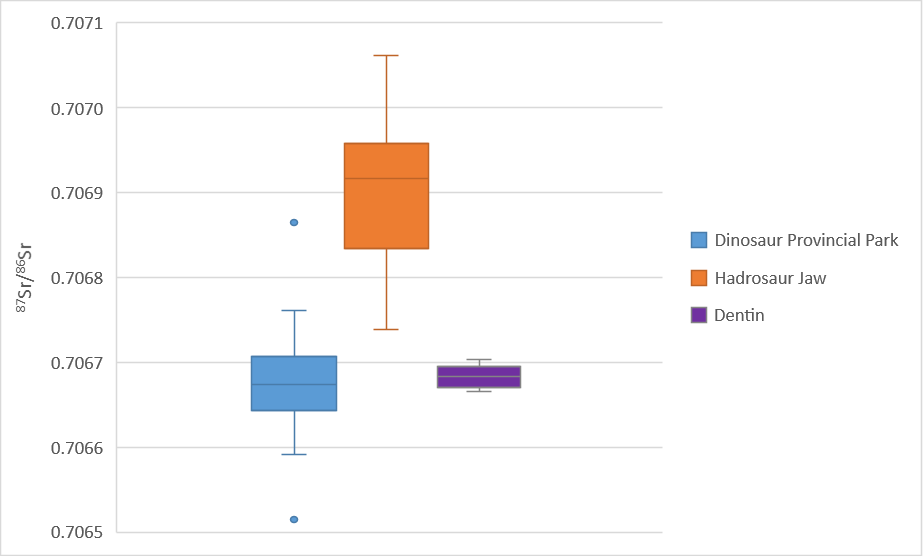
Figure S2: Comparison of dentin isotope ratios to enamel ratios from Dinosaur Provincial Park. While only a few measurements of dentin are made, they are extremely consistent in comparison to enamel samples. Three samples collected from different positions in one tooth from the hadrosaur jaw, an isolated hadrosaur tooth and an isolated fish scale.

Statistical Analysis.

As mentioned in text, a Tukey statistical analysis was performed to determine the likelihood that each locality had a distinct isotopic signature based on the data. It was also used to determine the probability that the hadrosaur jaw is representative of any particular location. The details of this analysis can be seen in Figure S3. The results show a very high probability that each locality possesses a unique isotopic signature, and that the hadrosaur jaw does not belong to any single locality.

In addition, we applied a mixing model in a similar fashion to that seen in Brennen et al., 2015 [50]. This involves applying a discriminant function analysis to predict what percentage of the signal from the hadrosaur jaw could be explained by each of the localities in the study. This was done in R using the MASS package, by applying a linear discriminant analysis. The results show ~30% of the hadrosaur teeth could be generated by the SSR signal, while ~70% could be generated by the DPP signal.


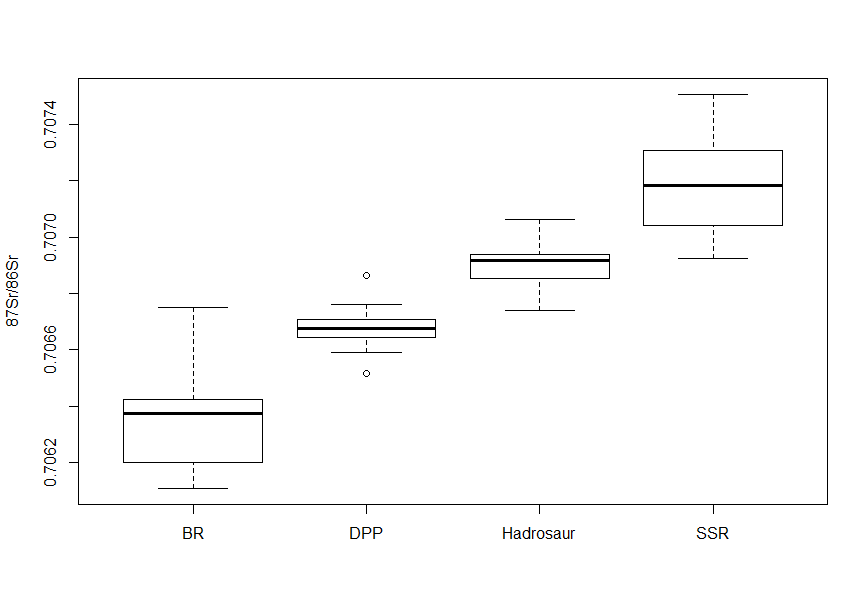


Figure S3: Box and whisker plots of data collected from the Battle River locality (BR), Dinosaur Provincial Park locality (DPP), the isolated hadrosaur jaw, and the South Saskatchewan River locality (SSR). The y-axis is the ^87^Sr/^86^Sr ratio. A Tukey Test comparing these four groups estimates the probability the hadrosaur jaw falls into either the DPP, BR or SSR localities, or neither. The results suggest that each group is distinct from all others, and the hadrosaur jaw does not belong to any other group (p<0.001). We have interpreted this to mean that some teeth in the jaw record the DPP signal, some the SSR signal, and others a mixed signal. This is consistent with an animal that moves between the two localities. Tukey Results: DPP-BR (p=5*10^-7^), DPP-SSR (p=0), SSR-BR (p=0), DPP-Hadrosaur (p=1.36*10^-5^), SSR-Hadrosaur (p=6.1*10^-6^), BR-Hadrosaur (p=0).

**Materials**

Materials used in this study included fossils collected in the field from Dinosaur Provincial Park in Alberta, Canada. These fossils were collected under the permit number 17-154. All material is either stored at the University of Calgary or the Royal Tyrrell Museum. Full details of accession number and storage for each fossil in this study is in Table S2.

| Accession Number | Storage Facility | Sample ID for this study |
| --- | --- | --- |
| UC 18126 | UofC | J-1 through J-20 |
| UC 18127 | UofC | 100-1 through 100-7 |
| UC 18128 | UofC | 146-1, 146-2, 146-3, 146-4, 146-6, 146-7 |
| TMP95.157.1 | RTMP | MBB-7, MBB-8 |
| TMP95.157.35 | RTMP | MBB-4, MBB-5, MBB-6 |
| TMP95.157.5 | RTMP | MBB-1, MBB-2, MBB-3 |
| TMP96.62.9 | RTMP | HAS-1, HAS-2 |
| TMP96.77.11 | RTMP | HAS-3, HAS-4, HAS-5 |
| TMP2000.021.0005 | RTMP | SSR-6 |
| TMP2000.021.0006 | RTMP | SSR-1, SSR-2, SSR-3, SSR-12, SSR-14, SSR-15 |
| TMP2000.021.0007 | RTMP | SSR-4, SSR-5, SSR-7, SSR-8, SSR-10, SSR-13 |
| TMP2019.060.0056 | RTMP | BR-5.1, BR-5.2, BR-5.2.1 |
| TMP2019.060.0059 | RTMP | BR-2 |
| TMP2019.060.0060 | RTMP | BR-1.1, BR-1.2, BR-1.3, BR-1.4 |
| TMP2019.060.0063 | RTMP | BR-4.1, BR-4.2, BR-4.3, BR-4.3.1 |
| TMP2019.060.0064 | RTMP | BR-3 |
| TMP2020.060.0001 | RTMP | H-1, H-2, H-3, H-4, H-5 |
| TMP2020.060.0002 | RTMP | T-1, T-2, T-3 |
| TMP2020.060.0003 | RTMP | M-1, M-2, M-3, M-4 |
| TMP2020.060.0004 | RTMP | G-1, G-2, G-3, G-4 |
| TMP2020.060.0005 | RTMP | C-1, C-2, C-3, C-4 |

Table S2: Table of specimen accession numbers and storage facility. Abbreviations are as follows: UofC = University of Calgary, RTMP = Royal Tyrrell Museum of Paleontology.

**References**

41. McArthur, JM, Howarth, RJ, Shields, GA 2012 Strontium isotope stratigraphy. In *The Geologic Timescale 2012* (Ed Gradstein, F.M., Ogg, J.G., Schmitz, M.D., Ogg, G.M.), 127-144. Elsevier

42. Braman DR. 2018 Terrestrial palynostratigraphy of the Upper Cretaceous (Santonian) to lowermost Paleocene of Southern Alberta, Canada, *Palynology* **42**, 102-147

43. van Hinsbergen, DJJ, de Groot, LV, van Schaik, SJ, Spakman, W, Bijl, PK, Sluijs, A, Langereis, CG, Brinkhuis, H. 2015 A paleolatitude calculator for paleoclimate studies (model version 2.1), *Plos One* **10**, e0126946

44. Eberth DA. 2005 The Geology, Currie PJ and Koppelhus EB eds In *Dinosaur Provincial Park: A Spectacular Ancient Ecosystem Revealed*. 1^st^ Edition, Indiana University Press, Bloomington, 54-82

45. Budd P, Montgomery J, Barreiro B, Thomas RG. 2000 Differential diagenesis of strontium in archaeological human dental tissues, *Applied Geochemistry* **15**, 687-694

46. Nelson BK, DeNiro MJ, Schoeninger MJ, De Paolo DJ, Hare PE. 1986 Effects of diagenesis on strontium, carbon, nitrogen and oxygen concentration and isotopic composition of bone, *Geochemica et Cosmochemica Acta* **50**, 1941-1949

47. Goodwin MB, Grant PG, Bench G, Holroyd PA. 2007 Elemental composition and diagenetic alteration of dinosaur bone: Distinguishing micron scale spatial and compositional heterogeneity using PIXE*, Palaeogeography, Palaeoclimatology, Palaeoecology* **253**, 248-276

48. Jeffree RA, Markich SJ, Twining JR. 2001 Element concentrations in the flesh and osteoderms of Estuarine crocodiles (*Crocodylus porosus*) from the alligator rivers region, Norther Australia: Biotic and Geographic effects, *Archives of environmental contamination and toxicology* **40**, 236-245

49. Shahnazari M, Sharkey NA, Fosmire GJ, Leach RM. 2009 Effects of Strontium on Bone Strength, Density, Volume, and Microarchitecture in Laying Hens, *Journal of bone and mineral research* 21, 1696-1703

50. Brennan SR, Zimmerman CE, Fernandez DP, Cerling TE, McPhee MV, Wooller MJ. 2015 Strontium isotopes delineate fine scale natal origins and migration histories of Pacific Salmon, *Science Advances* 1, e1400124
